# Operando Characterization of Electrochemistry at the Rutile TiO_2_(110)/0.1 M HCl Interface Using Ambient Pressure XPS

**DOI:** 10.1021/acs.jpcc.4c07173

**Published:** 2024-11-26

**Authors:** Jiangdong Yu, Conor Byrne, Jameel Imran, Zoë Henderson, Katherine B. Holt, Alexander I. Large, Georg Held, Alex Walton, Geoff Thornton

**Affiliations:** † London Centre for Nanotechnology and Chemistry Department, University College London, 20 Gordon Street, London WC1H 0AJ, U.K.; ‡ Department of Chemistry, University of Manchester, Manchester M13 9PL, U.K.; § Photon Science Institute, University of Manchester, Manchester M13 9PL, U.K.; ∥ Chemistry Department, University College London, 20 Gordon Street, London WC1H 0AJ, U.K.; ⊥ Diamond Light Source, Harwell Campus, Didcot OX11 0DE, U.K.

## Abstract

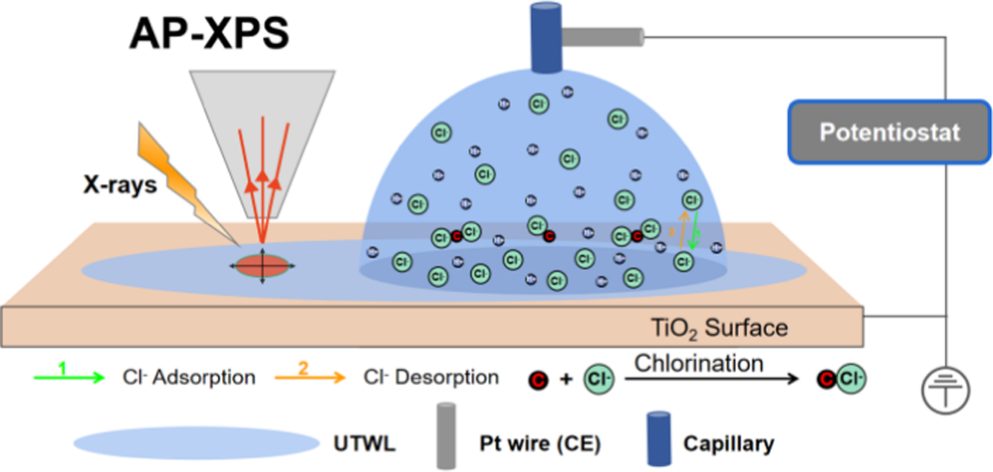

Ambient pressure X-ray photoelectron spectroscopy (AP-XPS) was employed to investigate the effect of applied potential on the interface of TiO_2_(110) with 0.1 M HCl. The study, which involved operando electrochemical characterization, enabled real-time monitoring and analysis of electrochemical processes. There is a significant influence on the interface composition; in particular, the surface Cl^–^ surface coverage varies with electrochemical potential. Moreover, there appears to be a reaction of evolved Cl with adventitious carbon to form C–Cl and C–Cl_2_ species.

## Introduction

Titanium dioxide (TiO_2_) is a widely used material that has a variety of applications. Of relevance to the current work is its photocatalytic properties.^1−4^ These have been explored extensively since the first observation of photoelectrocatalytic water splitting over TiO_2_ was demonstrated.^1^ Most work has been carried out on the photocatalytically active P25 powder, which is a mixture of the anatase and rutile polymorphs.^4^ Fundamental studies of the surface properties have focused on model single crystal surfaces of anatase and rutile, particularly rutile TiO_2_(110).^5^ Here we investigate this surface and its electrochemical interface with 0.1 M HCl. HCl is chosen as a model electrolyte based on knowledge of the structure of its interface with TiO_2_(110)^6^, as well as interest in the ability of Cl^–^ to enhance the photocatalytic yield of water splitting.^4^

Ultrahigh vacuum (UHV) is commonly used in surface chemistry studies to provide a controlled environment that eliminates the interference of impurities and gases in the analysis of the surface. UHV (ca. 1 × 10^–10^ mbar) also provides a suitable environment for a number of surface analysis techniques, such as X-ray photoelectron spectroscopy (XPS). However, one of the challenges in understanding the details of photocatalysis is that reactions occur at the solid/liquid interface. More recently, ambient pressure XPS (AP-XPS) has been developed, which readily allows the study of surfaces and interfaces at pressures of ∼20 mbar and above.^7^ This is close to the vapor pressure of water at room temperature, making the study of solid interfaces with aqueous solutions possible using AP-XPS.^8,9^ Moreover, employing a tunable X-ray source allows depth profiling of the interface. In previous work, we demonstrated the use of an offset-droplet method^10^ in an AP-XPS study of the TiO_2_(110) interface with ultrathin water wetting layers.^11^

Here, we explore the use of the offset-droplet method for in-operando AP-XPS studies of electrochemical interfaces. Such work has been explored previously using a so-called dip and pull technique^8,12^ as well as a hybrid approach that has aspects of the dip and pull and offset droplet methods.^13^ The offset droplet method has the advantage of employing a nonvertical sample geometry, providing more stability, and is compatible with a variety of sample sizes. This allows for the ready integration of UHV sample preparation methods. The interface of TiO_2_(110) with 0.1 M HCl is examined in-operando, with the results demonstrating the utility of the method that points to future application in photocatalytic studies.

## Experimental Section

A newly reduced n-type rutile TiO_2_ (110) single crystal sample (Pi-Kem) was prepared using a modified version of a previously reported wet chemical preparation method that is known to result in a well-ordered (1 × 1) surface termination.^14^ In brief, the steps in the process were, in order, sonication in acetone, ethanol, and deionized water, followed by 90 min of air annealing at 973 K. Subsequently, the sample was placed in an ozone cleaner for 20 min to reduce C contamination.

**Figure 1:**
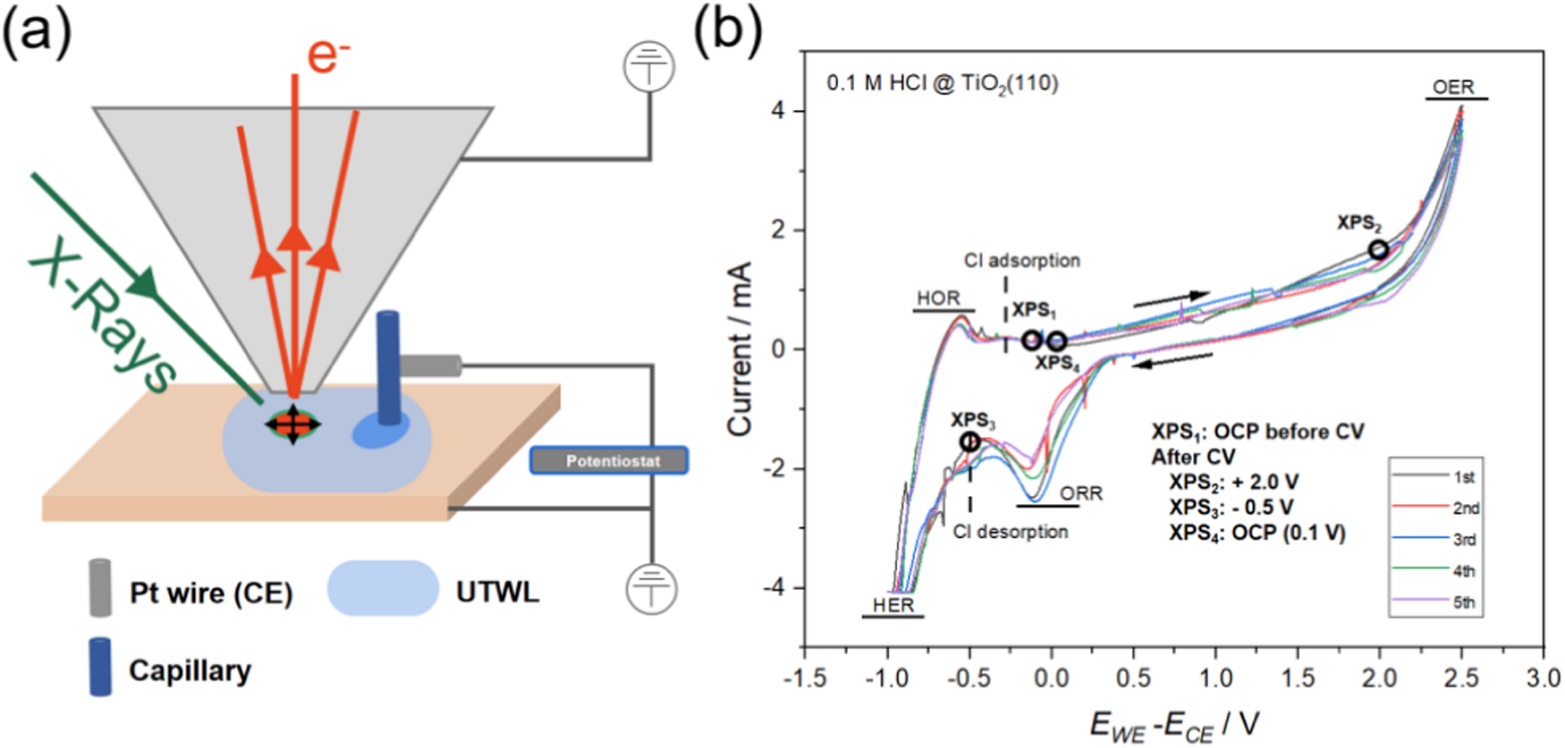
Experimental setup and electrochemical characterization. (a) Schematic of the experimental apparatus with the two-electrode electrochemical arrangement; (b) cyclic voltammetry (20 mV/s) for the TiO_2_ electrode in 0.1 M HCl (OCP: open circuit potential; HER: hydrogen evolution reaction; HOR: hydrogen oxidation reaction; OER: oxygen evolution reaction; ORR: oxygen reduction reaction). Prior to and following CV measurements, XPS data were collected at different bias voltages (XPS_1_: OCP before CVs; XPS_2_: +2.0 V; XPS_3_: −0.5 V; XPS_4_: OCP (0.1 V)).

Experiments were performed using the ambient-pressure photoemission end-station on VerSoX (B07) at Diamond Light Source (250 ≤ *h*ν ≤ 2800 eV).^15^ This end-station is equipped with a differentially pumped hemispherical Phoibos 150 NAP electron energy analyzer, where the analysis chamber is backfilled to ambient pressures. In these measurements we employed a so-called offset droplet method to examine solid/liquid interfaces, as described in previous publications.^10,11^ For the electrochemical measurements an Ivium potentiostat was used to control the potential of electrodes in a solution. Here the sample acts as the working electrode, which is grounded to the spectrometer. A platinum wire is attached to the side of the droplet capillary tube such that it is wet by the droplet. This serves as the counter electrode (CE) in this two-electrode system. A schematic of the two-electrode setup, with the sample in front of the electron energy analyzer front cone, is shown in Figure 1a. The sample was cooled to about 284 K using an external chiller before introducing droplets to minimize the background vapor pressure during AP-XPS analysis.

The sample was introduced at a base pressure of 5.0 × 10^–^^7^ mbar, when “as-loaded” spectra were recorded. Spectra were then recorded after the water partial pressure was increased to ∼13 mbar by introducing an ultrapure water (resistivity = 18.2 MΩ) droplet adjacent to the photoanalysis area, and then adjusting the sample position to examine an ultrathin water layer (UTWL). For the electrochemical measurements, the 0.1 M HCl electrolyte (pH 1) was prepared by diluting aqueous HCl (*Sigma-Aldrich*) with ultrapure water and then degassing the electrolyte by bubbling He gas to remove air bubbles that can affect the electrochemical measurements.

In these normal emission measurements, monochromatic radiation was incident at 30° with respect to the surface normal, with the (horizontal) electric vector in the [11̅0] azimuth. The system has a nominal energy resolution of 0.5 eV full-width at half-maximum (FWHM).^15^ Both IgorPro Ver 6 and CasaXPS peak fitting software programs were employed for the XPS peak fitting analysis. The line shapes were fitted using Voigt profiles along with Shirley backgrounds. To ensure consistency in peak fitting parameters, the same line shapes and peak widths were used for a given core level across all the sample data sets. Spectra were calibrated to the Ti 2p_3/2_ line at 459.5 eV to ensure consistent peak alignment across different electrochemical potentials. While peak shifts were observed during the measurements, these shifts are influenced by various factors in the electrochemical environment.^16^ A detailed view of the Ti 2p and C 1s spectra under different potentials is presented in Figure S1, providing evidence of the system’s in-operando electrochemical behavior during the AP-XPS measurements. Spectra are not normalized, unless specified otherwise. Since the photon flux is nominally constant at a given photon energy, intensities are modified only by chemical changes and photoelectron attenuation by vapor/liquid above the interface. Photon energies of 1000 eV, 1487 and 2000 eV were employed. These were shown in our earlier work^11^ to provide photoelectron escape depths large enough to observe the water/solid interface with a droplet thickness of about 10 nm. Moreover, 1487 eV was used for most of the work since it corresponds to the photon energy of Al K_α_, for which photoionization cross sections are readily available.^17^ The coverage of carbon on the as-loaded surface was estimated using the relative areas of the Ti 2p_3/2_ and C 1s peaks normalized to results from previous work.^18^ From this, the coverage of Si was estimated from the relative Al K_α_ cross sections of C 1s and Si 2p.^17^

## Results and Discussion

As-loaded TiO_2_ (110) was studied as a reference, followed by AP-XPS measurements at 13 mbar of water as well as ultrathin wetting layers of pure water and 0.1 M HCl. The electrochemical behavior was then investigated by recording cyclic voltammograms (CVs) of a UTWL 0.1 M HCl @ TiO_2_ (110) at a scan rate of 20 mV/s (see Figure 1b). Five cycles were recorded, with only small differences between them being observed. Overall, most of the information contained in the CV’s is related to the electrochemical reaction of water. As shown in Figure 1b, sweeping forward to the positive (anodic) potentials, the current increases above 2.0 V, attributed to the oxygen evolution reaction (OER).^19^ There may also be some contribution to the current from the chlorine evolution reaction (CER), which takes place at similar potentials.^20^

Reversing the voltage sweep direction results in the oxygen reduction reaction (ORR), with the peak at −0.11 V. Going to more negative (cathodic) potentials, the current of the hydrogen evolution reaction (HER) starts flowing from −0.7 V. Reversing the voltage sweep direction from negative to positive results in a return to open circuit potential after passing through the region of the hydrogen oxidation reaction (HOR). This corresponds to the peak at about −0.56 V, assigned based on its similarity with a feature observed for TiOx-Pt/C electrodes in 0.5 M HClO_4_.^21^ It is notable that the use of a two-electrode setup in this investigation leads to an increased separation between the OER and HER peaks. This observation is consistent with results obtained from earlier AP-XPS electrochemical experiments.^19^ The slight variations in peak separation between our study and prior observations stem from factors such as the influence of pH and the internal resistance drop across the TiO_2_ sample. Although most of the current signal comes from the electrochemical reaction of water, features can be discerned corresponding to Cl^–^ adsorption/desorption^22^ at about −0.28 V and −0.51 V, respectively.

Turning to the photoemission results, wide-scan, Ti 2p, O 1s and C 1s spectra of TiO_2_(110) recorded following the introduction of the sample (as-loaded), water vapor, and an UTWL of water are shown in Figures S2 and 2–4, respectively. Based on the relative humidity of 95%, the thickness of the water layer under water vapor is estimated to be 1.6 nm (8 molecular layers).^23^ The additional thickness arising from the UTWL is 8.5 nm, as estimated using the decrease in the Ti 2p signal and the 4 nm inelastic mean free path of water at 1000 eV.^11^ As shown in Figure S2, the as-loaded survey scan evidences a contribution from surface adsorbed adventitious carbon. Removing such a carbon layer with wet chemical preparation is possible,^14^ although this was not pursued here since the study aimed to achieve a model interface closer to that in a real-world electrochemical cell. As well as 0.1 ML of C, the presence of 0.05 ML (1 Monolayer is defined as the number of substrate surface unit cells) of Si contamination is also evidenced on the as-loaded surface; Si has previously been observed in the end-station. Water vapor introduction is accompanied by a decrease in count rate, as expected from attenuation of the photoemitted electrons, with a further decrease following UTWL formation.

**Figure 2:**
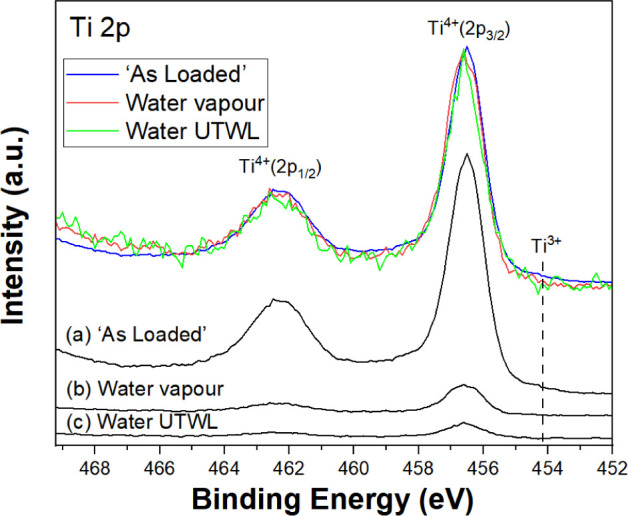
Ti 2p AP-XPS (*h*ν = 1487 eV) from TiO_2_(110) for (a) as loaded, (b) in a background pressure of 13 mbar of water, and (c) covered with a 10 nm thick layer of pure water. Overlaid spectra are normalized to the Ti 2p_3/2_ signal.

The Ti 2p core level XPS spectra shown in Figure 2 exhibit a small Ti 2p_3/2_ shoulder peak at a binding energy of 457.6 eV, likely corresponding to a minimal concentration of residual subsurface Ti^3+^ states.^24^

**Figure 3:**
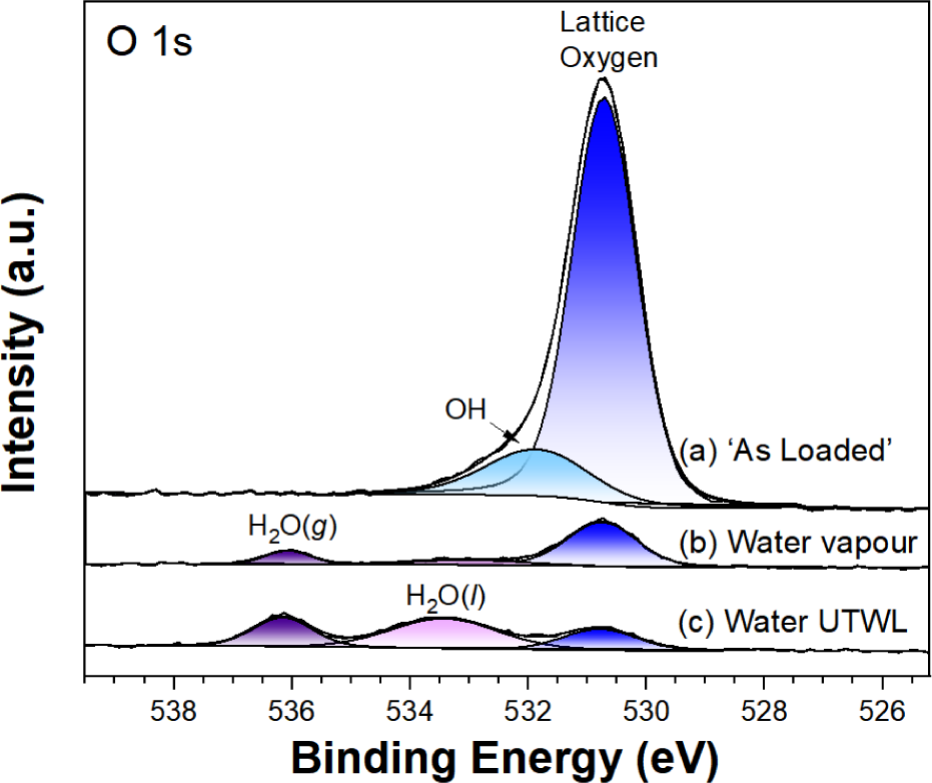
O 1s AP-XPS (*h*ν = 1487 eV) from TiO_2_(110) for (a) as-loaded, (b) water vapor, and (c) water UTWL. The fits to Voigt peak shapes along with the Shirley backgrounds are shown.

Regarding the O 1s spectrum of the as-loaded sample in Figure 3a, the feature at a binding energy (BE) of 530.7 eV corresponds to the lattice oxide peak for TiO_2_. There is also a higher binding energy shoulder (531.8 eV), which arises from OH surface groups.^23^ Since C–OH species are evidenced in the C 1s spectra shown below, an O 1s contribution will appear at a similar binding energy from the surface OH.^25^ When exposed to water vapor, the O 1s spectrum (Figure 3b) also shows changes as evidenced by the appearance of a peak at 535.7 eV BE, which arises from gas phase H_2_O between the sample and analyzer cone.^26^ In order to examine the solid/water interface, the analysis area was moved to the UTWL. As seen in Figure 3c, this exhibits a peak at 533.2 eV from H_2_O(*l*). For all three interfaces, the intensity of the OH feature relative to the lattice oxygen peak remains unchanged, consistent with previous studies.^23^

**Figure 4:**
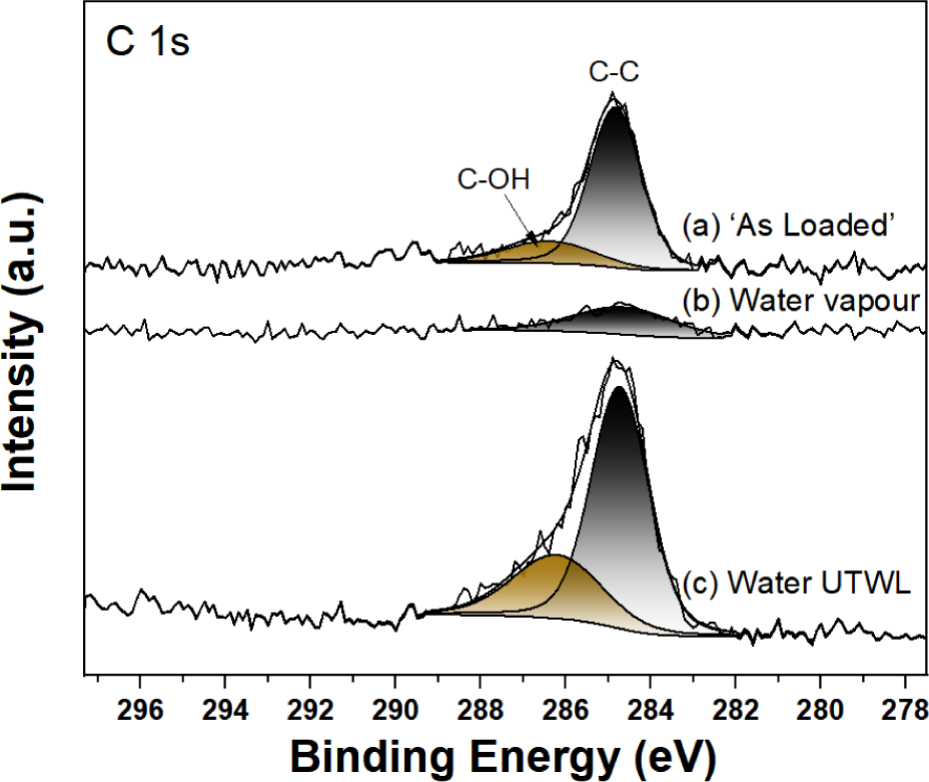
C 1s AP-XPS (*h*ν = 1487 eV) from TiO_2_(110) for (a) as loaded, (b) water vapor, and (c) water UTWL. The fits to Voigt peak shapes along with the Shirley backgrounds are shown.

Based on previous work,^27^ the as-loaded spectrum in Figure 4a evidence C–C bonds at 284.6 eV, with a lower contribution from C–OH and/or C–O–C bonds at 286.4 eV. Introduction of water vapor attenuates the signal (Figure 4b),^15^ while the formation of the UTWL leads to an increase in the C–C and C–OH intensities (Figure 4c). Such an increase could have a contribution from X-ray beam effects.^28^ There is no indication of carboxylate groups, which would lie at about 289 eV, consistent with earlier XPS results.^11,29^

**Figure 5:**
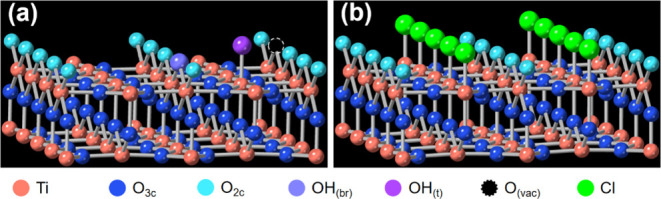
Ball-and-stick models of (a) the as-prepared TiO_2_ surface and (b) the TiO_2_(110)/0.1 M HCl contact layer expected from SXRD measurements.^5,6,18^

Figure 5 shows the ball-and-stick model of the expected structure for a wet chemistry prepared rutile TiO_2_ (110) surface,^5,18^ with the titanium atoms represented by red spheres and the oxygen atoms represented by blue spheres. The surface contains five-coordinated titanium atoms, two-coordinated oxygen atoms, and bridging oxygen atoms, along with representative functional groups. The model also includes oxygen vacancies, which are represented as empty sites where oxygen atoms are missing. When immersed in water, surface X-ray diffraction (SXRD) data evidence the formation of 0.5 ML terminal OH.^18^ However, at the TiO_2_(110)/0.1 M HCl interface Cl replaces OH at the Ti_5c_ site, with 1 ML coverage.^6^ As noted above, the CV measurement results evidence the desorption (−0.51 V) and adsorption (−0.28 V) of chloride from the surface at different points in the electrochemical cycles.

**Figure 6:**
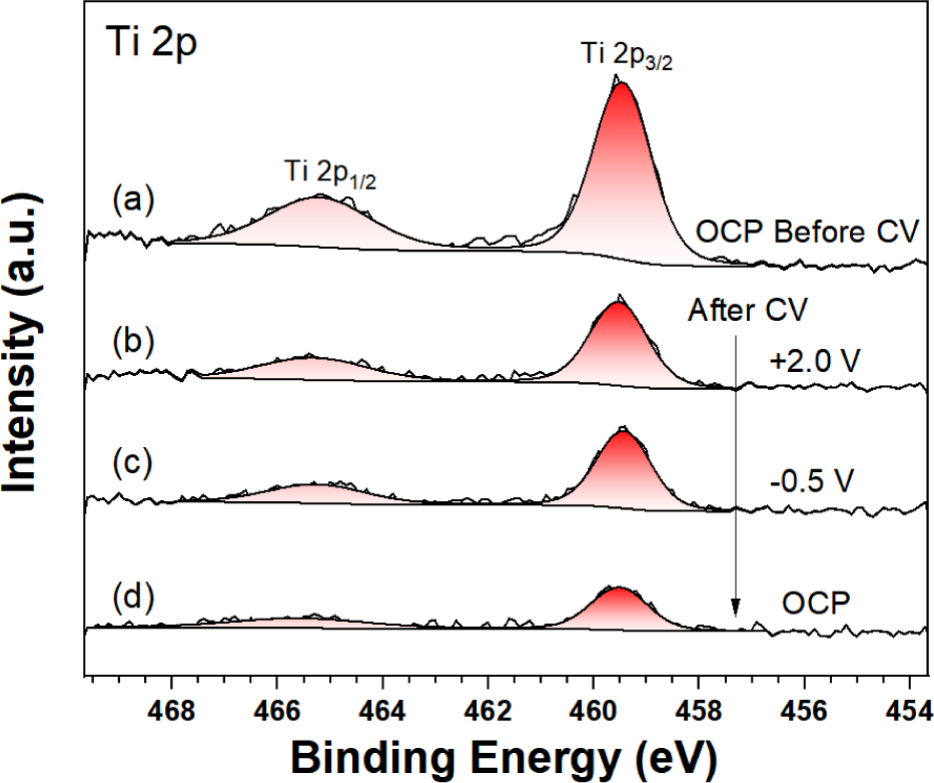
Ti 2p AP-XPS (*h*ν = 1487 eV) from TiO_2_(110)/0.1 M HCl before CVs and after CVs at different potential biases: (a) OCP before CVs, (b) +2.8 V, (c) +0.3 V, and (d) OCP. The fits to Voigt peak shapes along with the Shirley backgrounds are shown.

**Figure 7:**
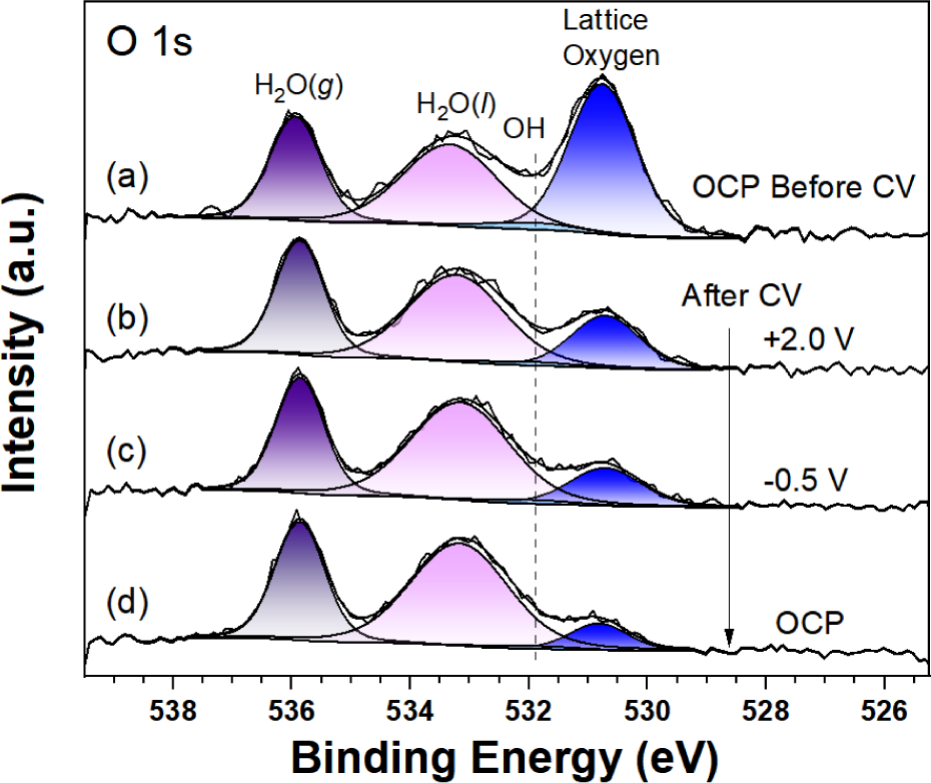
O 1s AP-XPS (*h*ν = 1487 eV) from TiO_2_(110)/0.1 M HCl before CVs and after CVs at different potential biases: (a) OCP before CV, (b) +2.8 V, (c) +0.3 V, and (d) OCP. The fits to Voigt peak shapes along with the Shirley backgrounds are shown.

In order to monitor the electrochemical changes of the surface species on the TiO_2_ electrode, AP-XPS data from Ti 2p, O 1s, C 1s and Cl 2p regions were collected at different electrochemical potentials. These are compared with spectra recorded from the 0.1 M HCl UTWL interface with TiO_2_ (110) before the CV measurements. The thickness of the electrolyte UTWL is calculated to be 3.5 nm, using the attenuation of the Ti 2p signal. Figure 6 shows the development of the Ti 2p peak for the TiO_2_ (110) working electrode during the electrochemical measurement. The decrease in the signal of Ti and bulk O peak (seen in Figure 7) intensity is likely due to a fluctuation in the electrolyte layer thickness caused by electrowetting effects. There will also be an added contribution from photoelectron attenuation resulting from the higher carbon and Cl coverage following the CV measurements. The absence of any traces of Ti^3+^ or Ti^2+^ signals in the Ti 2p spectra indicates that the electrochemical treatment had no impact on the near-surface stoichiometry of rutile.

AP-XPS O 1s spectra obtained at varying electrochemical potentials are shown in Figure 7, where the spectra are fitted to peaks corresponding to four species. The peak at 530.7 eV is attributed to lattice oxygen, while a small shoulder at 531.8 eV is ascribed to OH surface groups, whereas the features located at 533.2 and 535.7 eV correspond to liquid water and gas phase water, respectively.^26^

**Figure 8:**
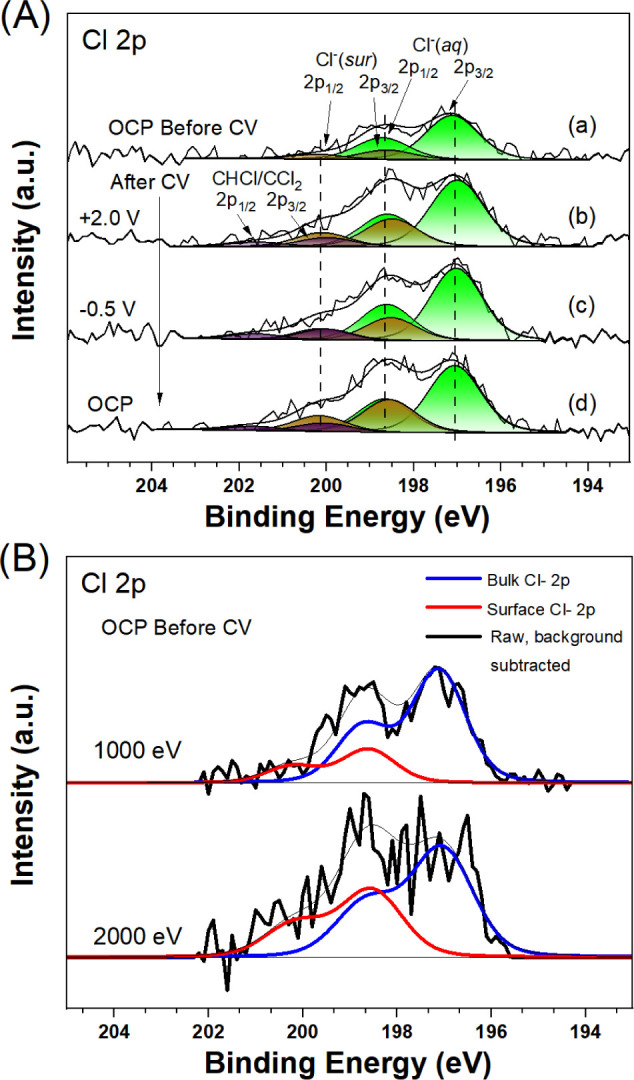
(A) Cl 2p AP-XPS (*h*ν = 1487 eV) from TiO_2_(110)/0.1 M HCl before CVs and after CVs at different potential biases: (a) OCP before CV, (b) +2.8 V, (c) +0.3 V, and (d) OCP; (B) photon depth profiling at 1000 and 2000 eV, where spectra are normalized to the bulk (aqueous) Cl 2p_3/2_ peak. The fits to Voigt peak shapes are shown along with the Shirley backgrounds in (A).

To confirm the presence of the Cl adsorbates, Cl 2p spectra (Figure 8) were investigated during the electrochemical measurement. Each spectrum consists of three spin–orbit doublets (Cl 2p_1/2_-2p_3/2_): the first Cl 2p_3/2_ at 197.1 eV is attributed to the Cl^–^ ions in the bulk electrolyte; the second Cl 2p_3/2_ peak at 198.6 eV is attributed to surface Cl adsorbates at the Ti_5c_ site (see Figure 8A), which is assumed to have a coverage of 1 ML at OCP before the CV measurements based on previous work;^6^ the third Cl 2p_3/2_ peak at 200.2 eV is attributed to the formation of covalent chlorine C–Cl species. There is a 1.6 eV spin–orbit splitting between the 2p_3/2_ and 2p_1/2_ peaks of each of these species, and the region under the 2p_3/2_ peak is twice that under the 2p_1/2_ peak. For each species, the full width at half-maximum (FWHM) of the 2p_3/2_ and 2p_1/2_ peaks is constrained to be equal (∼1.5 eV). Before CV measurements, recordings were made for photon energy depth profiling at 1000 and 2000 eV, as shown in Figure 8B. The higher binding energy peak is relatively more intense in the 2000 eV measurement. Since the photoemission penetration depth is higher at 2000 eV, this indicates that the higher BE species arises from interface Cl^–^, with the lower BE component corresponding to Cl^–^(aq). This assignment is consistent with previous work on bromide species.^30^ The assignment of the third species of Cl to C–Cl is based on previous XPS studies.^31 −34^

After the CV measurements, Cl 2p spectra recorded at +2.0 V show that the amount of surface Cl adsorbates increased significantly. However, the Cl^–^(sur) signal intensity decreased when the applied bias voltage was shifted to −0.5 V, which is consistent with the CV results (Figure 1b) that Cl adsorption occurs around −0.28 V and desorption occurs around −0.51 V. Also consistent with this, the interface features return to the higher level in the spectrum recorded at OCP after the CV. Given the assumed 1 ML coverage of Cl before electrochemistry, then the increase in coverage after the CV measurements implies surface restructuring or etching to increase the surface area. Figure S3 shows the variation of the Cl coverage in more detail.

**Figure 9:**
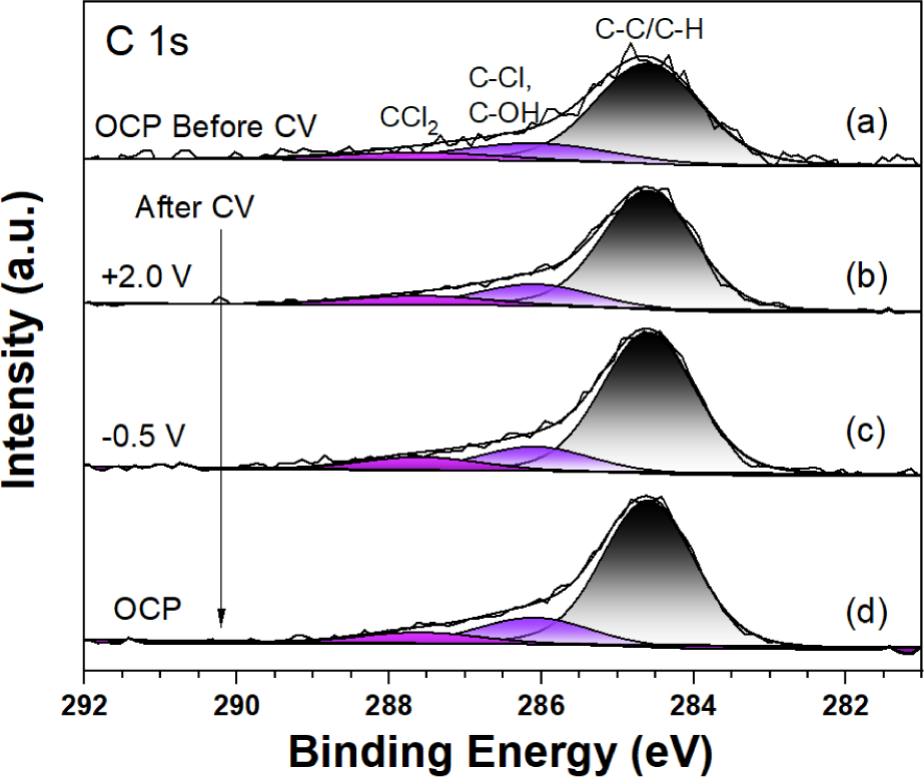
AP-XPS (*h*ν = 1487 eV) C 1s of TiO_2_(110)/0.1 M HCl before CVs and after CVs at different potential biases: (a) OCP before CVs, (b) +2.8 V, (c) +0.3 V, and (d) OCP. The fits to Voigt peaks along with the Shirley backgrounds are shown.

Finally, in Figure 9, the C 1s core-level spectra are presented. Notably, there is a gradual increase in the concentration of adventitious carbon observed over the course of the electrochemical measurements. Based on previous work,^31^ the three distinctive components within the spectrum can be assigned as follows: CC/CH_2_ at 284.6 eV, CHCl at 286.1 eV, and CCl_2_ at 287.6 eV. There could also be a contribution from C–OH at 286.4 eV. It is clear that aliphatic carbon C–C/C-H dominates the C 1s spectral region. Furthermore, our experimental data yields a noteworthy observation. The peak intensities of C–Cl and C–Cl_2_ exhibit a pronounced increase after the CVs, consistent with the Cl 2p results. This surprising behavior is attributed to the desorption of Cl^–^ ions from the TiO_2_ surface, and their subsequent bonding to adventitious carbon. Such a role of adventitious carbon in the electrochemistry has not previously been evidenced as far as we are aware and could play an important role in the interface chemistry. In this connection, it is of interest to note that chlorination of sp^3^-H bonds has been employed in organic synthesis.^35^ As seen in Figure S3b, the coverage of carbon steadily increases throughout the progression of the experiment. This is accounted for by the combined increase of C–Cl and C–Cl_2_ (Figure S3c,d), pointing to further electrochemical chlorination of adventitious carbon.

There is no detectable carboxylate species in the C 1s spectra, which would appear at 289 eV. This might have been expected, at least in the case of the water UTWL, based on the conclusions of earlier work.^36^ However, the results from a more recent study^29^ are in line with our observations here.

## Summary

This study demonstrated the successful use of AP-XPS to perform in-operando electrochemical characterization based on the offset droplet method, with in situ analysis of Cl^–^ ions at the TiO_2_(110) solid/liquid interface at various bias voltages. The results show the presence of different species of Cl on the surface. They also evidence the adsorption/desorption of chloride ions from the surface during the electrochemical process. It was also noted that the electrolyte layer grown in situ was contaminated with adventitious carbon, but no evidence of carboxylate carbon species was found. Overall, the study provides valuable insights into the behavior of Cl^–^ ions on the TiO_2_ surface under different conditions. It also highlights the potential of AP-XPS in the study of solid/liquid electrochemical interfaces involving well-characterized single crystal substrates. The observation of electrochemical chlorination of adventitious carbon following electrochemistry was unanticipated, suggesting that further work is warranted.

## Supporting Information Available

Additional AP-XPS results; the coverages of Cl(sur), total C, CCl, and CCl_2_ under different electrochemical conditions, along with a discussion of their origin (PDF)
