# Supplementary material for: Operando Characterization of Electrochemistry at the Rutile TiO2(110)/0.1 M HCl Interface Using Ambient Pressure XPS
Source: J Phys Chem C Nanomater Interfaces. 2024 Nov 26;128(49):20933–9. doi: 10.1021/acs.jpcc.4c07173 (PMC11647877; doi:10.1021/acs.jpcc.4c07173)
Supplement: Supplementary file 1 — jp4c07173_si_001.pdf [file jp4c07173_si_001.pdf]

Supplementary Information

Operando Characterization of Electrochemistry at the Rutile TiO_2(110)/0.1 M HCl Interface Using Ambient Pressure XPS

Jiangdong Yu[1], Conor Byrne[2,3], Jameel Imran[1], Zoë Henderson[2,3@], Katherine B. Holt[4], Alexander I. Large[5], Georg Held[5], Alex Walton[2,3*] and Geoff Thornton[1*]

1. London Centre for Nanotechnology and Chemistry Department, University College London, 20 Gordon Street, London WC1H 0AJ, UK
2. Department of Chemistry, University of Manchester, Manchester M13 9PL, UK
3. Photon Science Institute, University of Manchester, Manchester M13 9PL, UK
4. Chemistry Department, University College London, 20 Gordon Street, London WC1H 0AJ, UK
5. Diamond Light Source, Harwell Campus, Didcot, UK

E-mail: alex.walton@manchester.ac.uk, g.thornton@ucl.ac.uk

Present address:

@ NSG Group, NSG Technology Centre, Hall Lane, Lathom, Ormskirk, Lancashire L40 5UF, UK

Supplementary Information Includes:

Fig. S1, S2; Additional AP-XPS results

Fig. S3; Coverages of Cl(sur), total C, CCl and CCl_2 under different electrochemical conditions, along with a discussion of the origin of their behavior.

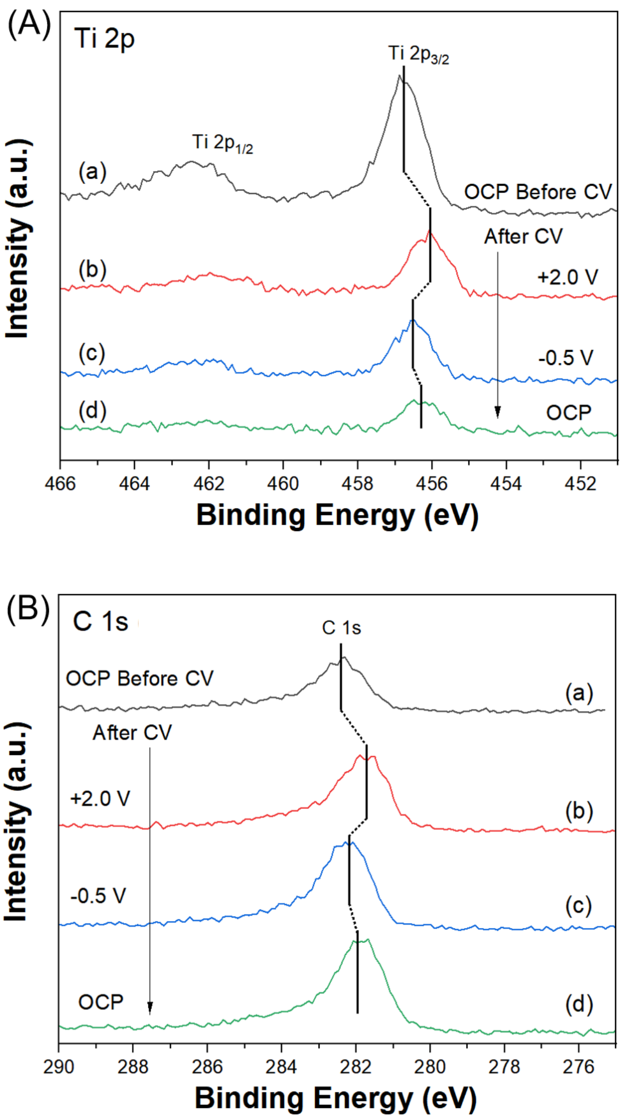

Figure S1 (A) Ti 2p and (B) C 1s AP-XPS (hν = 1487 eV) from TiO_2(110)/0.1 M HCl before CVs and after CVs at different potential bias (a) OCP before CV, (b) +2.8 V, (c) +0.3 V and (d) OCP. The changes in Ti 2p and C 1s signal reflect the system's response to applied potential as well as the effect of the combined resistance of sample and droplet, demonstrating electrochemical activity despite the complex factors influencing peak positions during reactions[1].

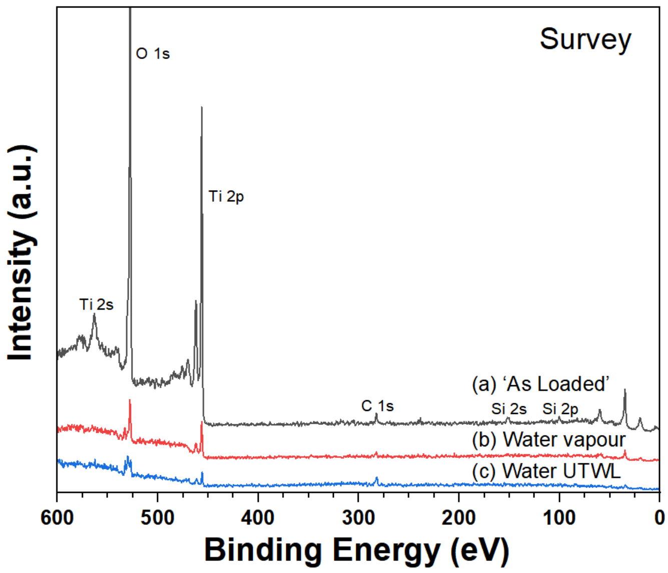

Figure S2 AP-XPS survey spectra (hν = 1487 eV) of TiO_2 (110) as-loaded, and in a background pressure of 13 mbar water as well as covered with a 10 nm thick layer of pure water.

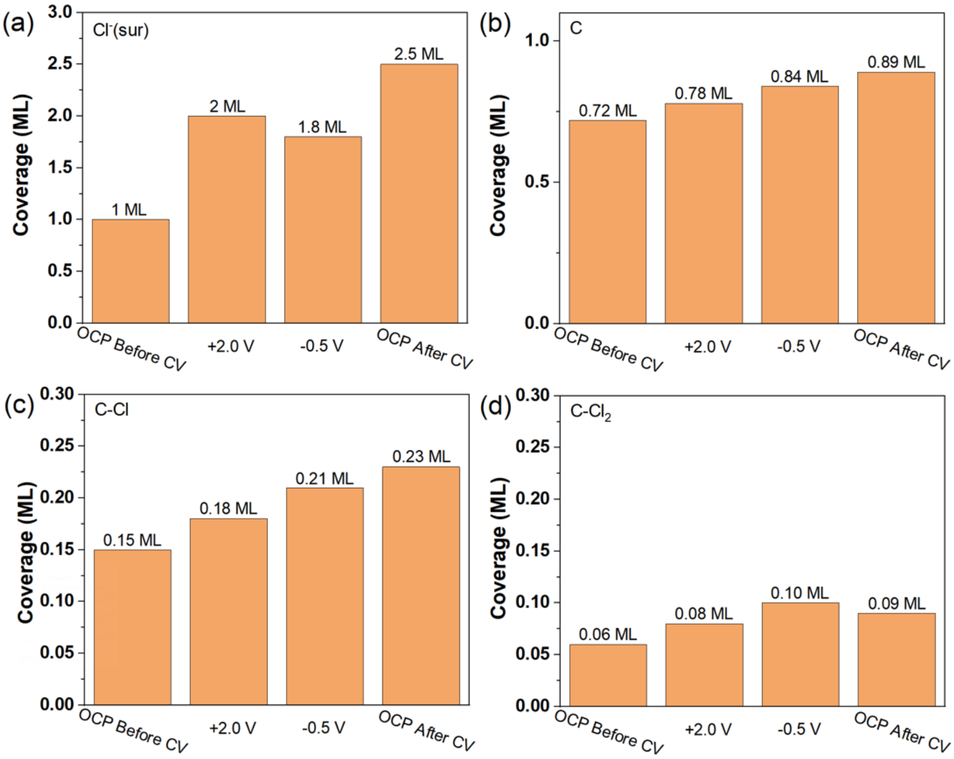

Figure S3: Changes in coverage of different species on the TiO_2 (110) surface during electrochemical treatment in 0.1 M HCl using XPS intensities. (a) Cl^-(sur) from the Cl $2p_{3/2}$ peak area; (b) total C from the C 1s area, (c) C-Cl, and (d) C-Cl_2 taken from the corresponding Cl $2p_{3/2}$ peak area. In calculating the coverages, we assume that the coverage of Cl^- at OCP before CV is 1 ML[2]. The other coverage values were calculated by normalizing spectra to the Ti $2p_{3/2}$ peak area to minimize the effect of changing electrolyte thickness, with the C coverages calculated using the relative cross sections. This assumes that the attenuation through the electrolyte is the same for the Ti, Cl and C core levels, and neglects coverage of Cl^-(sur) by C species.

References

1. Teschner, D.; Plescher, J.; Piccinin, S; Jones, T.E.; Hammud, A.; Schmidt, F.; Knop-Gericke, A.; Bluhm, H.; Shavorskiy. Understanding anomalous gas-phase peak shifts in dip-and-pull ambient pressure XPS experiments. *J. Phys. Chem. C* **2024**, 128, 7096-7105.
2. Nadeem, I.M.; Penschke, C.; Chen, J.; Torrelles, X.; Wilson, A.; Hussain, H.; Cabailh, G.; Bikondoa, O.; Imran, J.; Nicklin, C.; et al. Ultra-compact electrical double layers at TiO_2(110) electrified interfaces. *J. Am. Chem. Soc.* DOI: 10.1021/jacs.4c09911
